# Enzymatically Modified Fats Applied in Emulsions Stabilized by Polysaccharides

**DOI:** 10.3390/biom11010049

**Published:** 2020-12-31

**Authors:** Magdalena Woźniak, Małgorzata Kowalska, Serge Tavernier, Anna Żbikowska

**Affiliations:** 1 Faculty of Chemical Engineering and Commodity Science, Kazimierz Pulaski University of Technologies and Humanities, Chrobrego 27, 26-600 Radom, Poland; 2 iPRACS–Intelligence in Processes, Advanced Catalysts and Solvents, Faculty of Applied Engineering, University of Antwerp, Groenenborgerlaan 171, B-2020 Antwerp, Belgium; serge.tavernier@uantwerpen.be; 3 Department of Food Technology and Assessment, Institute of Food Sciences, Warsaw University of Life Sciences-SGGW (WULS-SGGW), 02-772 Warsaw, Poland; anna_zbikowska@sggw.pl

**Keywords:** lipids, emulsions, enzymatic modification, xanthan gum, sclerotinum gum

## Abstract

The subject of the study was emulsions based on enzymatically modified fats and stabilized with polysaccharides (xanthan gum and scleroglucan). Emulsion oil phases (blends of mutton tallow and hemp seed oil in a ratio of 3:1, 3:2, 3:3, 2:3 and 1:3) were characterized in the terms of acid value, melting point and mono- and diacylglycerols content before and after the modification. Emulsions containing modified fat blends and various amount (0.6, 0.8 and 1.0% *w/w*) of polysaccharides were investigated in the terms of their color, rheological properties, microstructure, droplet size and stability. The obtained results confirmed that enzymatic modification allowed to produce new fats, which can successfully be applied as an emulsion oil phases equipped with a sufficient amount of emulsifiers. The use of a variable amount of texture modifier in the proposed formulations did not show clear differences in the stability of the systems. Therefore, it does not seem justified to use greater amounts of a modifier (above 0.6% *w/w*) in this type of emulsions. The proposed formulations could be of interest to the cosmetics, food or pharmaceutical industry.

## 1. Introduction

Lipids are chemically diverse group of organic compounds [1]. Their physiological and biochemical roles are essential to the human body [2]. Due to their unique properties, these biomolecules are of great importance particularly in food sector, although they found application also in pharmaceutical, cosmetic and chemical industries [3]. Fats and oils, that belong to simple lipids, are derived from animal or vegetable matter, and in their natural form are not always ideal for the industrial utilization [4]. Thus, they are often subjected to various modification processes to improve their properties as well as nutritional value in order to increase their potential use [4,5]. One of the modification techniques, which allows to improve the functionality of fats and oils and to create a low or zero trans product is enzymatic interesterification [6,7]. This technique utilizes lipase catalysis and thus has a number of advantages over chemical interesterification. These include environmental considerations, such as fewer processing and product purification steps, lower energy consumption due to milder processing conditions, but also some economic aspects [4,8].

The most common recent applications of enzymatically interesterified fats include margarins, shortenings, butter equivalents and infant formulas [9]. Another possibility is the use of such modified fats in oil-in-water emulsions [10]. Due to the appropriate design of the parameters of enzymatic reaction, it is possible to shift the equilibrium of the interesterification reaction towards the hydrolysis. As a result, a certain planned amount of by-products such as mono- and diacylglycerols (MAG and DAG) will be formed in the final product, which can be directly used as emulsifiers in emulsion systems. In our previous study [10] we have performed the optimization of water concentration in the reaction environment, which allowed to form sufficient amount of emulsifiers in the fat blend to stabilize the emulsion system. MAG and DAG obtained by this kind of process, are characterized by a specific differentiation and uniqueness of their chemical structure depending on, among others, type and ratio of the fats used as substrates for a reaction. 

In the study, we have decided to produce the fat bases of emulsion systems with built-in emulsifiers derived from mutton tallow and hemp seed oil. Mutton tallow, which is a waste of the meat industry, in relation to other animal fats, is characterized by a relatively high content of conjugated linoleic acid (CLA), which contributes to the benefit of human health [11]. Due to the significant predominance of saturated fatty acids, mutton tallow has high oxidative stability. However, as a product of animal origin it has a negligible content of polyunsaturated fatty acids (PUFA) [12]. In order to enrich this fat with PUFA, it was enzymatically modified with hemp seed oil, the main fatty acids of which are linoleic acid (omega-6) and α-linolenic acid (omega-3) [13]. Moreover, this oil, due to the content of γ-linolenic acid, is a desirable component of preparations applied to the skin [13].

Emulsifiers, together with other substances stabilizing emulsion systems, i.e., texture modifiers, act through different stabilization methods to improve the stability of emulsions [14]. Therefore, it is important to obtain information on the compatibility of the emulsifiers formed during the enzymatic interesterification with texture modifiers. For this study, we have chosen a commercial texture modifier containing two natural polysaccharides: xanthan gum and sclerotinum gum (scleroglucan). Xanthan gum is a product of aerobic fermentation performed by bacterium *Xanthomonas campestris*, whereas sclerotinum gum is produced as a result of the same process by fungus *Sclerotium rolfsii* [15,16]. Polysaccharides are well known thickeners applied in emulsions for various purposes, due to their effectiveness, non-toxicity, biocompatibility, and biodegradability [14]. Especially popular in food, cosmetic and pharmaceutical sector is xanthan gum, its unique chemical structure influence obtaining favorable rheological properties as well as pseudoplasticity of the products in which this substance is applied [16]. 

The aim of the study was to prepare oil-in-water emulsions containing as an oil phase enzymatically modified blends of mutton tallow and hemp seed oil stabilized with polysaccharides (xanthan gum and scleroglucan). The work also aimed at characterization of formulations properties and stability with respect to the concentration of texture modifier as well as the ratio of fats in modified fat blends served as oil phases. The novelty of the study is related to the formulation of emulsions containing emulsifiers derived from the enzymatic process with the combination of the polysaccharides applied. The topic is rather poorly investigated in the scientific literature so far, so this study brings a basic information in this area.

## 2. Materials and Methods 

### 2.1. Material

Mutton tallow (MT) was donated by Meat-Farm (Stefanowo, Wólka Kosowska, Poland), hemp seed oil (HSO) (Oleofarm, Wrocław, Poland) was purchased in local market. As a reaction catalyst lipase from *Rhizomucor miehei* immobilized on immobead 150, ≥300 U/g (Sigma-Aldrich, Zwijndrecht, The Netherlands) was used. As a texture modifier for emulsions, commercial product Actigum VSX 20 (Cargill, Krefeld, Germany) was used, containing sclerotium gum and xanthan gum. As preservative Euxyl K712 (Schülke & Mayr GmbH, Norderstedt, Germany) was used, which was an aqueous solution of sodium benzoate and potassium sorbate.

### 2.2. Methods for Fats Preparation and Analysis

#### 2.2.1. Enzymatic Modification of Fats

Mutton tallow was bleached and deodorized before the reaction, according to the procedure described in our previous study [7]. Fat blends were prepared by mixing MT and HSO in the following ratios: 3:1, 3:2, 3:3, 2:3 and 1:3 (*w/w*). Then the blends were placed in a shaker equipped with a water batch (SWB 22N, Labo Play, Bytom, Poland) and thermostated at 60 °C for 15 min. The reaction was started after addition of an immobilized lipase from *Rhizomucor miehei* (5.0% *w/w* in relation to the fat blend mass) and distilled water (1.1% *w/w* in relation to the fat blend mass) to the fat blends. Water concentration in the reaction environment was dictated by previously performed optimization [10]. The reaction was performed at 60 °C for 6 h with 200 rpm shaking speed, and was stopped by filtering the enzyme.

#### 2.2.2. Acid Value

The acid value (AV) was determined by manual titration according to ISO 660:2009 method [17]. The results were presented as a mean value of 3 determinations ± standard deviation (SD).

#### 2.2.3. Melting Point

The melting point (MP) was determined in open capillary tubes according to ISO 6321:2002 method [18]. The results were presented as a mean value of 3 determinations ± standard deviation (SD).

#### 2.2.4. DAG and MAG Content

DAG and MAG content was determined by means of Agilent 1100 series GPC system. Chromatographic conditions in detail were described in our previous study [7]. The results were presented as a mean value of 3 determinations ± standard deviation (SD).

### 2.3. Methods for Emulsions Preparation and Analysis

#### 2.3.1. Emulsion Preparation

Aqueous phases of the emulsions were prepared by dispersing a respective amount (Table 1) of texture modifier—Actigum VSX 20 in distilled water by means of magnetic stirrer (IKA, RCT Basic, Staufen, Germany) for 30 min, then the phases were homogenized for 1 min at 18,500 rmp using a ULTRA-TURRAX, model T18 equipped with S18G–19G dispersing head (IKA, Shanghai, China) and left for 24 h. Oil phases consisted of modified MT and HSO fat blends in a various ratios (according to Table 1). Both phases were heated to 50–55 °C, then blended and homogenized for 4 min at 18,500 rmp using a ULTRA-TURRAX, model T18 equipped with S18G–19G dispersing head (IKA, Shanghai, China). After homogenization, emulsions were cooled to ambient temperature and the preservative was added.

#### 2.3.2. Color Assessment

Color assessment was performed using a chromameter CR-400 (Konica Minolta Sensing Inc., Milton Keynes, UK). CIEL*a*b* system was used, where: L* (lightness)—ranging from 0 (black) to 100 (white); a*—ranging from −60 to +60, (−) greenness, (+) redness, b*—ranging from −60 to +60, (−) blueness, (+) yellowness [16]. The following parameters were calculated [19]:

(1) $$C^{*}=\sqrt{a^{*}{}^{2}+b^{*}{}^{2}}$$

(2) $${\Delta E}^{*}=\sqrt{{\Delta a}^{*}{}^{2}+{\Delta b}^{*}{}^{2}+{\Delta L}^{*}{}^{2}}$$

(3) $$C^{*}{}_{\mathrm{after}\;\mathrm{storage}}\;=\left|C^{*}{}_{24h}-C^{*}{}_{30\;\mathrm{days}}\right|$$

where: C*—Chroma, ΔE*—total color difference, C*_24 h_–Chroma determined on freshly prepared (24 h at 2–7 °C) emulsions, C*_30 days_–Chroma determined on stored emulsions (30 days at 2–7 °C), C*_after storage_ – modulus of difference between C*_24 h_ and C*_30 days_. ΔE*_after storage_ was calculated analogically to the Equation (3).

According to Pathare et al. [19] changes in perceivable color can be classified as very distinct when ΔE > 3; distinct when 1.5 < ΔE < 3; small when 1.5 < ΔE. The determination was performed on freshly prepared emulsions (in the manuscript the term “freshly prepared” means 24 h after their preparation) and stored emulsions (30 days at 2–7 °C). The results were presented as a mean value of 3 determinations ± standard deviation (SD).

#### 2.3.3. Rheological Analysis

For rheological analysis of the freshly prepared emulsions, a rotational viscometer Brookfield DV-III Ultra, model HA with helipath spindle set (Brookfield Engineering laboratories, Stoughton, MA, USA) was used. The measurement was performed using T-bar spindle no. 93 (T-C) for the following speeds: 2.0, 5.0, 10.0, 15.0, and 20.0 rpm at 20 °C. For each speed, the apparent viscosity values (Pa*s) were recorded. Values are presented as a mean of three measurements.

#### 2.3.4. Stability of Emulsions 

The kinetics of destabilization processes were determined using Turbiscan^Lab^ (Formulaction, Toulouse, France). Turbiscan uses MLS (multiple light scattering) analysis, and the principle of determination is based on backscattering (BS) and transmission (T) light intensity variations. Stability of the emulsions was evaluated on a basis of Turbiscan Stability Index (TSI), which was calculated according to the following formula:

(4) $$\mathrm{TSI}=\underset{i}{\sum }\frac{\Sigma_{h}\left|{\mathrm{scan}}_{i}\left(h\right)-{\mathrm{scan}}_{i-1}\left(h\right)\right|}{H}$$

where: scan_i_(h)—mean BS light intensity for _i_(h) scan at a given height h, scan_i−1_(h)—mean BS light intensity for the _I−1_(h) scan at a given height h, and H is the sample height [20]. 

TSI is a parameter that takes into account all the scans performed for a sample. Higher TSI values are related to less stable system [20].

#### 2.3.5. Microphotographs of Emulsions 

Microphotographs of the freshly prepared emulsions were taken using a Delta Optical Genetic Pro Trino (Delta Optical, Warsaw, Poland) microscope and a DLT-Cam PRO digital camera (Delta Optical, Warsaw, Poland) at ×10 and ×40 objective.

#### 2.3.6. Droplet Size 

Mean, minimum and maximum droplet size of the freshly prepared emulsions were determined by means of laser diffraction method, using MasterSizer 3000 (Malvern Instruments Ltd., Malvern, Worcestershire, UK) equipped with a wet sample dispersion unit (Malvern Hydro MV, Worcestershire, UK). Samples were diluted in distilled water (1.0 g of the sample in 20.0 mL) and sonicated for 16 min before the measurement. The refractive indexes of the dispersed phase and dispersing medium were set as 1.470 and 1.330, respectively. Mean droplet size was presented as the volume mean diameter D_(4,3)_. The results were presented as a mean value of 3 determinations.

#### 2.3.7. Statistical Analysis

One-way ANOVA was used for analyzing the experimental results and Tukey’s test were used to determine the significance between the means (*p* < 0.05). The Statistica 13 software (Statsoft, Cracow, Poland) was used.

## 3. Results and Discussion

### 3.1. Fat Blends Characteristics

**Figure 1:**
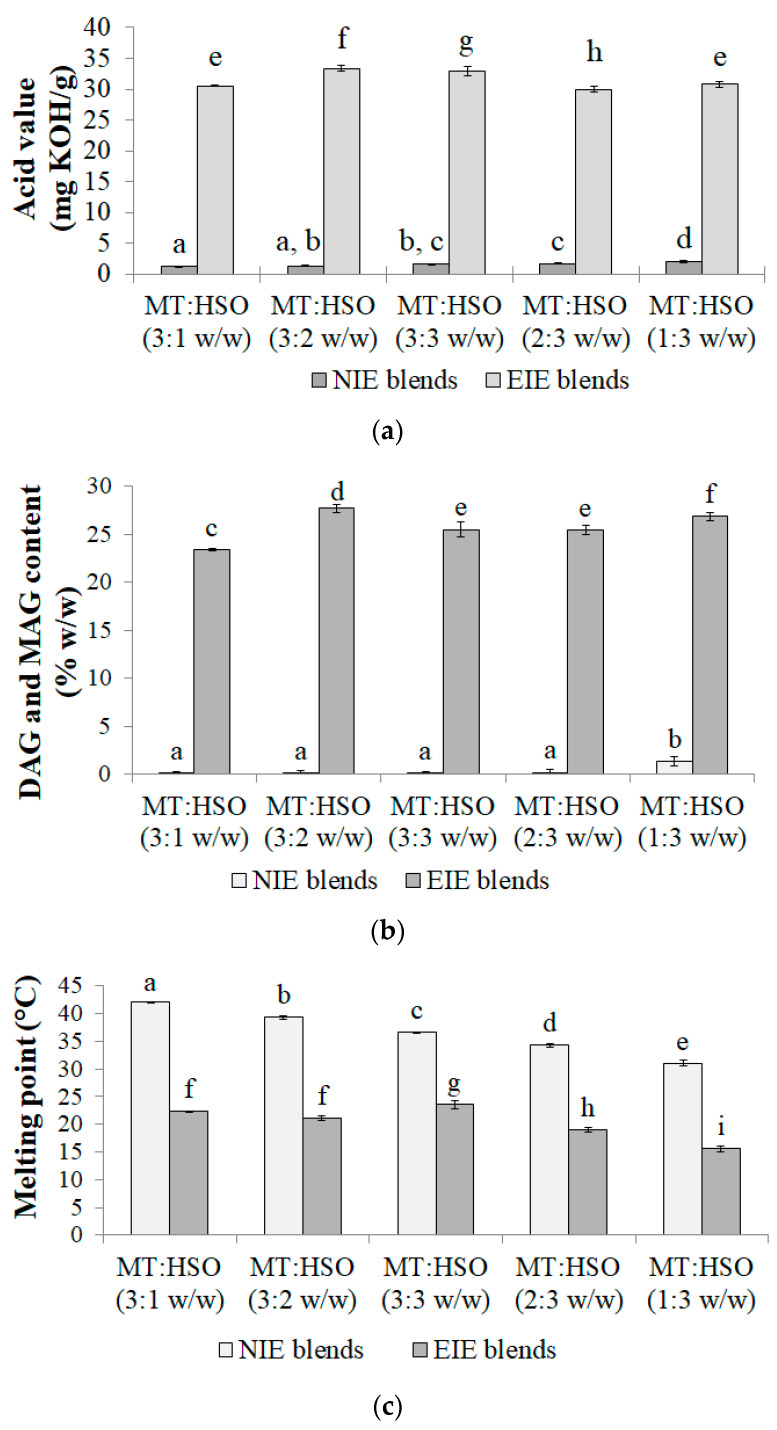
Fat blends characteristics: (**a**) acid value, (**b**) DAG and MAG content, (**c**) melting point. Different letters indicate significantly different values (*p* < 0.05).

In the study, the enzymatic interesterification with partial fat hydrolysis was performed. The obtained new fats, as well as their initial blends, were characterized in terms of their melting point, acid value and the content of mono- and diacylglycerols. Figure 1a shows that the enzymatic modification caused a significant (*p* < 0.05) increase in the acid value of the fat blends. There was no relationship observed between the ratio of MT and HSO in the fat blends and the acid value obtained. The acid value for the enzymatically modified (EIE) blends was in the range of 30.0–33.4, while for the non-modified (NIE) blends between 1.2 to 2.0. The acid value is related to the amount of free fatty acids present in a fat blend and at the same time indicates the content of other compounds of the polar fraction, i.e., mono- and diacylglycerols. In the enzymatic modification process, the addition of water to the reaction environment had an effect on the high acid value, although it was dictated by obtaining a greater amount of MAG and DAG in the final blends. In the study it was intended to shift the interesterification reaction equilibrium towards hydrolysis. Fat blends containing triacylglycerols and a sufficient amount of MAG and DAG can constitute a product with a ready-made emulsifier, the substance necessary to form a stable dispersion system. It was observed that the highest amount of mono- and diacylglycerols was obtained for the modified blend of MT:HSO in a ratio of 3:2 *w/w* and accounted for 27.7% *w/w* (Figure 1b). Comparable amounts of these components were determined for the EIE blends of MT:HSO in a ratio of 3:3 and 2:3 *w/w*, 25.5 and 25.4% *w/w* respectively (Figure 1b). The indicated values confirmed that the modification process occurred and changed the physicochemical properties of the newly formed fats.

Another parameter modified by the enzymatic reaction was the melting point. The melting points of non-modified blends tended to decrease with increasing proportion of hemp seed oil, due to the increase in the low melting point TAG content [21]. Fat blends after the enzymatic modification were characterized by significantly (*p* < 0.05) decreased melting points (Figure 1c). The lowest values, both before and after the reaction, were noted for MT:HSO fat blend in a ratio of 1:3 *w/w*. On the other hand, the fat blend of MT:HSO 3:1 *w/w* showed the greatest decrease in the melting point (by 19.8 °C). For the remaining blends, the decrease ranged from 13.0 to 18.3 °C. Xie and Zang [22] reported similar results and noted that the enzymatic modification of vegetable oil and animal fat allowed to obtain fat blends with decreased melting point in comparison with their initial blends.

### 3.2. Emulsions Characteristics

#### 3.2.1. Color Assessment

**Figure 2:**
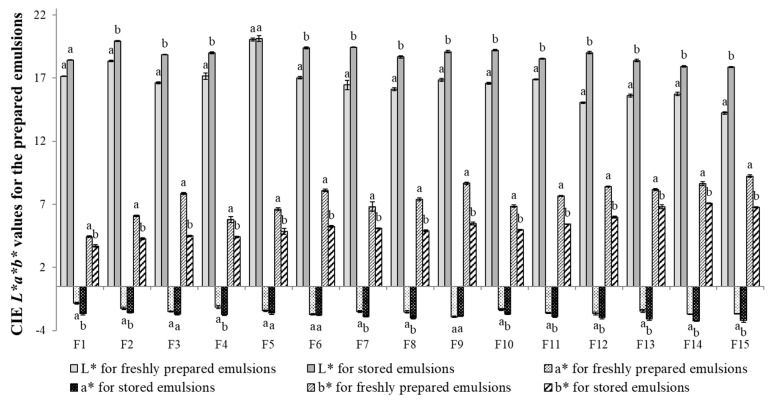
CIE L*a*b* values for the prepared emulsions. a, b—different letters for the same parameter for freshly prepared and stored emulsions indicate significantly different values (*p* < 0.05).

A particularly important task during the development of emulsion products is to maintain the characteristics of these products that are significant for consumers, which do not vary over a long period of time from the acceptable and expected level. Stability of dispersion systems is related to these expectations. The destabilization processes make the product highly unattractive for the consumers. Destabilization can manifest itself through a visible change in the color of the system. It was found that after the storage period, the L*, a* and b* parameters changed slightly (Figure 2). Considering the L* parameter, a slight increase for all emulsions was observed after storage suggesting a lightening of the color. Emulsion F15 was characterized by the darkest color after preparation (L* = 13.7) and for this emulsion, the highest increase in the L* parameter value (of 3.7) was observed. It should be noted, however, that the color changes between emulsions containing various texture modifier content were not observed visually, nor was confirmed by the obtained L* parameter values.

In relation to parameter a*, its values increased with increasing content of the texture modifier (i.e., F1–F3, F4–F6, F7–F9, F10–F12, F13–F15) for all the freshly prepared formulations (Figure 2). After the storage period, no apparent instability of this parameter was observed. A slight increase in this parameter towards deepening the green color was observed for all emulsions. However, similarly to the previously describe L* parameter, these changes were not visually detected. Referring to the results obtained for the b* parameter, a significant influence of texture modifier content as well as the share of the amount of oil in an emulsion oil phase was observed. With the increase in the amount of polysaccharides and hemp seed oil in the emulsions, b* value increased, which suggested a greater proportion of yellow color in these systems. Similar tendency was observed for emulsions after the storage period. However, a decrease in the value of this parameter for all stored emulsions was observed when compared to the freshly prepared ones, which could indicate changes towards a greater share of blue color. The greatest changes were noted for the F15 emulsion.

**Figure 3:**
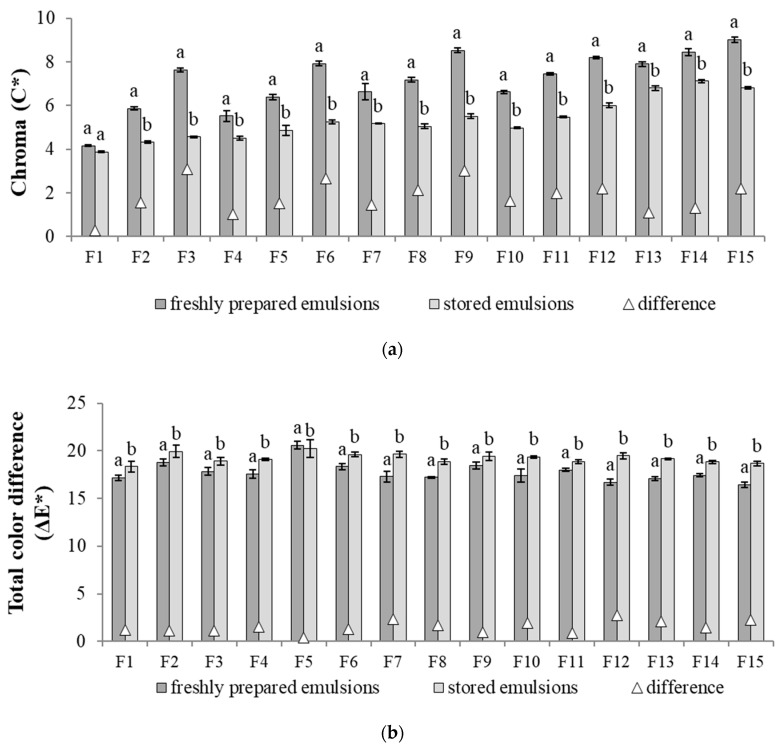
Emulsion color assessment (**a**) Chroma (C*); (**b**) Total color difference (ΔE*). a, b—different letters for the same parameter after storage period (i.e., C* for freshly prepared and C* for stored emulsions, etc.) indicate significantly different values (*p* < 0.05).

Referring to the value of the Chroma (C*) parameter, it was observed that with the increase of the texture modifier content (i.e., F1–F3, F4–F6, F7–F9, F10–F12, F13–F15) higher values of this parameter were noted (Figure 3a). The differences in color intensity between freshly prepared and stored emulsions were the greater, the higher the content of polysaccharides in the system. After the storage period, the F1 emulsion showed the smallest changes in the values of this parameter. On the other hand, if the type of oil phase was considered, it was found that the samples with the highest share of hemp seed oil were characterized by the smallest changes in this parameter resulting from the storage period (Figure 3a).

According to Patras et al. [23] total color difference (ΔE*) parameter can indicate the significance of color difference between stored and control samples. None of the analyzed emulsions showed significant (very distinct) changes after the storage period, defined by the above mentioned author as ΔE* > 3. The ΔE* ranged from 0.4 to 2.8 (Figure 3b), thus, based on the results obtained, it can be concluded that destabilization processes were not present in these systems.

#### 3.2.2. Rheological Analysis

**Figure 4:**
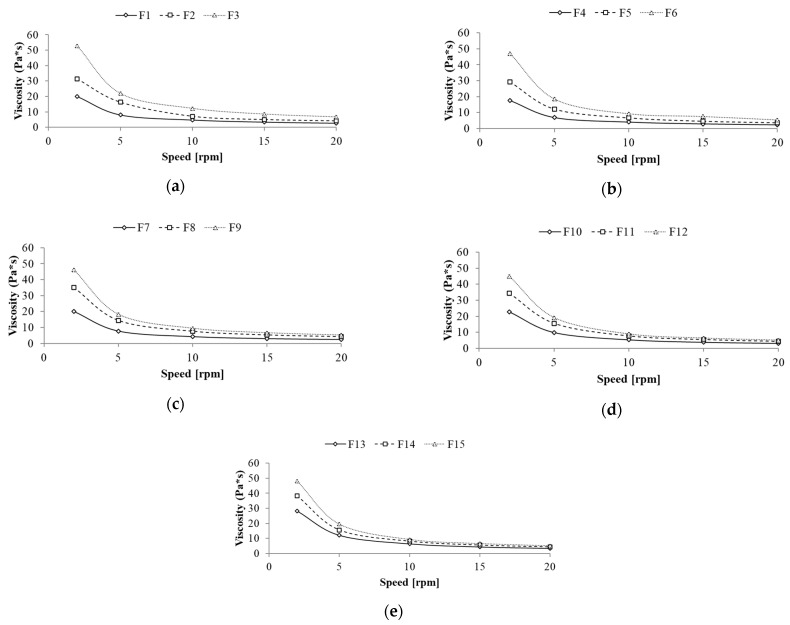
Rheological behavior of the freshly prepared emulsions with a following MT:HSO ratio in an oil phase: (**a**) 3:1 *w/w*, (**b**) 3:2 *w/w*, (**c**) 3:3 *w/w*, (**d**) 2:3 *w/w* and (**e**) 1:3 *w/w*.

Figure 4 shows the results of viscosity as a function of spindle speed (rheological behavior) of the prepared formulations. All formulations presented a pseudoplastic behavior. According to Estanqueiro et al. [24] for emulsion skin preparations pseudoplastic flow is the most common behavior, since pseudoplastic formulation can break down for easy spreading over the skin and the applied film can increase viscosity instantly to running resist.

The flow behavior of the prepared formulations was significantly influenced by the polysaccharides content. For all formulations increase in apparent viscosity values was noted with an increasing texture modifier content, i.e., F1 < F2 < F3; F4 < F5 < F6 etc. (Figure 4). Moreover, for emulsions containing modified fat blends with a predominance of mutton tallow in an oil phase (F1–F3 and F4–F5), these differences were greater, especially for the spindle speed of 2 rpm. For the subsequent spindle speeds (5, 10, 15, and 20 rpm), smaller variations in viscosity values for the same emulsions were observed, although the graphs do not show any tendency to overlap. On the other hand, this kind of tendency was observed for emulsions containing enzymatically modified fat with a predominance of hemp seed oil in an oil phase (F10–F12 and F13–F15) (Figure 4). The obtained results indicated that the ratio of fats in the fat blends used as an oil phase as well as the type of fat modification have a significant impact on the viscosity of the emulsion systems [25]. Zhang et al. [26] proved that enzymatic interesterification changes the solid fat content in the modified fat, thus changing its rheological properties. Moreover, according to Riberio et al. [27] the process of interesterification of fat molecules, also changes the crystalline structure of fats. The rearrangement of the fatty acids on the glycerol backbone of the triglyceride of oil and solid fat has a significant impact on their subsequent behavior in emulsion systems. In the presented systems, the emulsion containing a MT:HSO fat blend in a ratio of 3:1 *w/w* and the texture modifier content of 1.0% *w/w* was characterized by the highest viscosity values. For the spindle speed of 2 rpm and 20 rpm apparent viscosity were 52.9 Pa*s and 6.7 Pa*s, respectively. 

#### 3.2.3. Stability of Emulsions 

**Figure 5:**
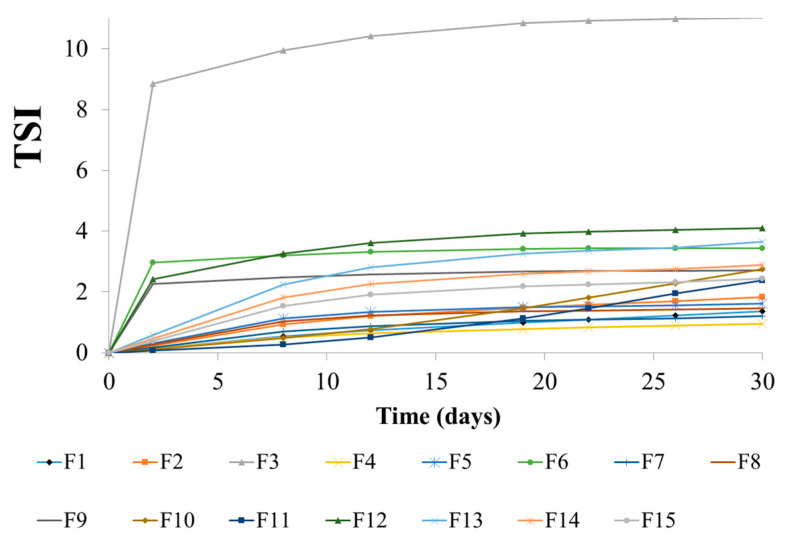
TSI values for the prepared emulsions during storage period.

The kinetics of emulsion destabilization processes was presented in Figure 5. It was observed that the F4 emulsion showed the greatest stability, i.e., for this emulsion the lowest values of TSI during the storage period were noted. The TSI for this emulsion did not exceed 0.95. However, it should be noted that for the remaining emulsions, apart from the F3, the TSI was on comparable level and did not exceed 4.10. The only emulsion with a significant increase of TSI was F3 emulsion, the value reached 11.0 after the storage period. The TSI values for F3 formulation was correlated with the highest droplet size noted for this system (Section 3.2.5). In the case of the emulsions containing oil phase with a higher proportion of mutton tallow, the values were lower than for emulsions containing oil phase with a higher proportion of hemp seed oil. 

#### 3.2.4. Microphotographs of Emulsions

**Figure 6:**
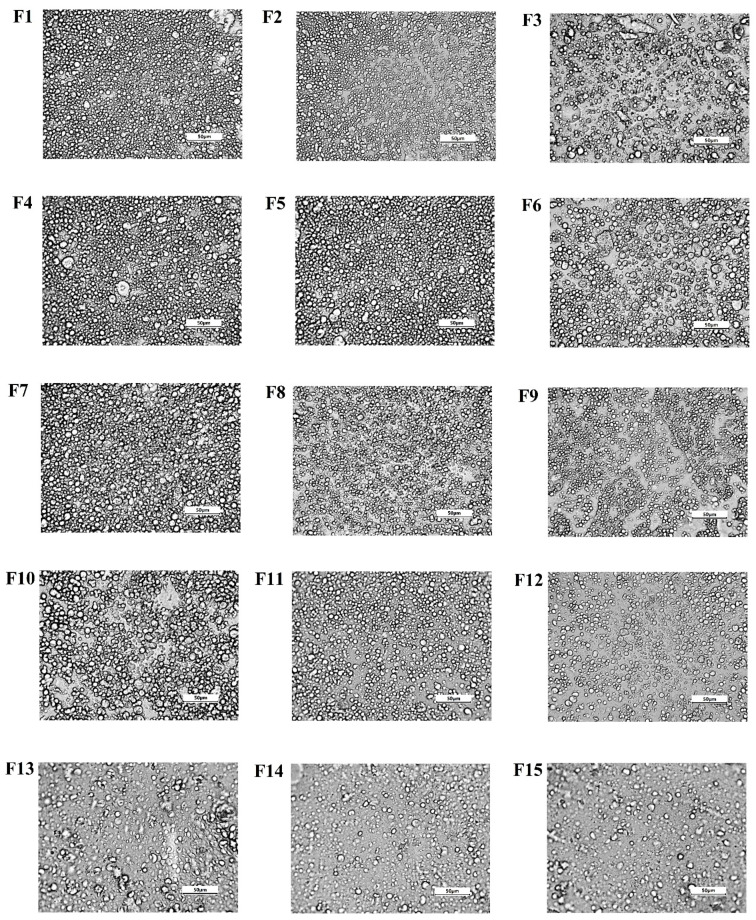
Microstructure of the freshly prepared emulsions. The bar corresponds to 50 µm.

**Figure 7:**
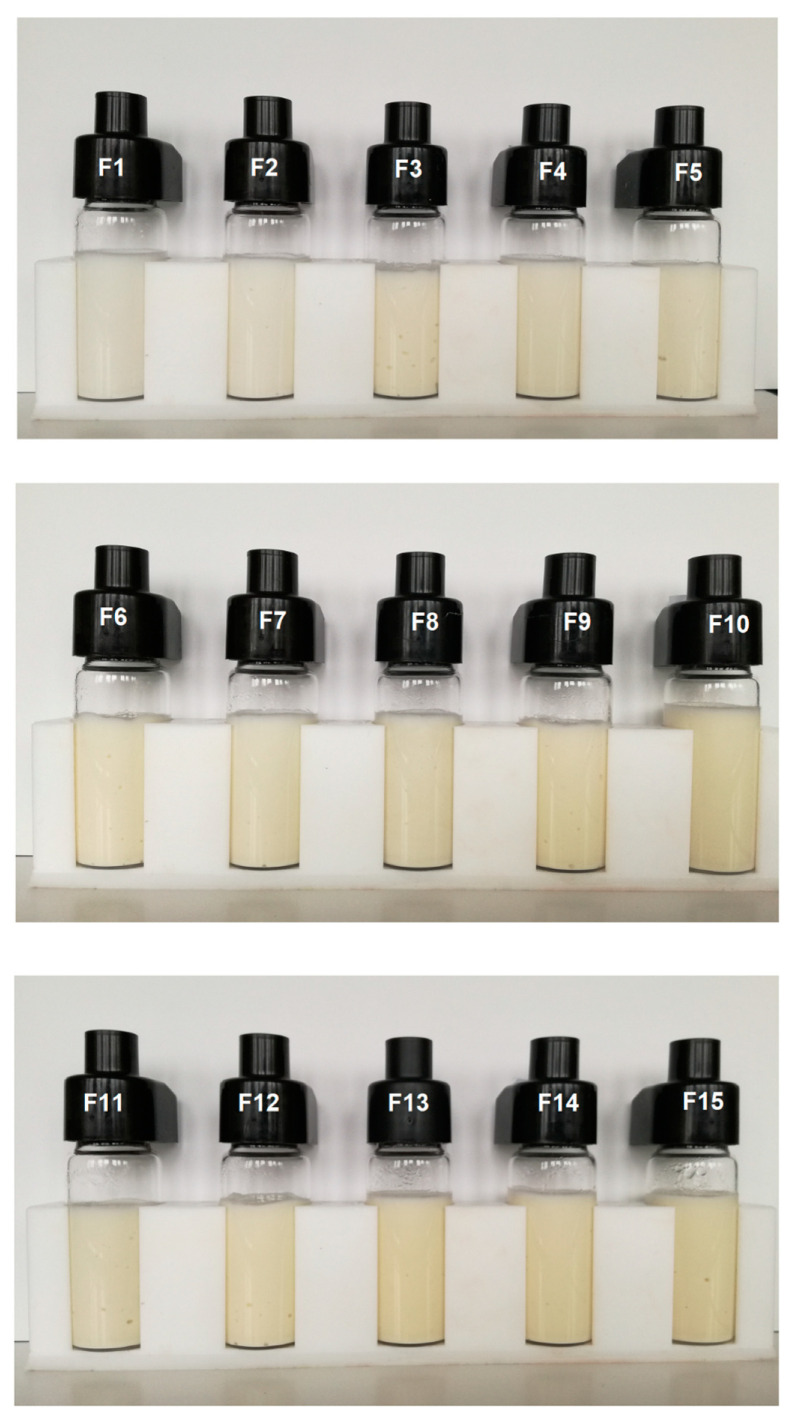
Photographs of the formulations after storage period.

The study of the microstructure of emulsion systems allows to assess the size and distribution of emulsion droplets, as well as the possible agglomeration processes. For almost all the emulsion systems produced, similar image and regular distribution of fat droplets were observed, which proves the homogeneous structure of these systems. Only one image (F3) showed irregularity and occasional clusters of agglomerated droplets (Figure 6). The above observations are also consistent with the visual assessment of the emulsions (Figure 7), which shows that after the storage period, the human eye could not register any destabilizing changes occurring in the systems.

#### 3.2.5. Droplet size of Emulsions

**Figure 8:**
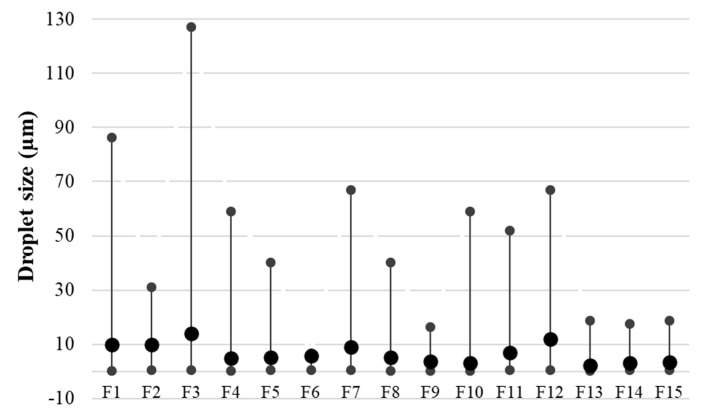
Minimum, mean and maximum droplet size of the freshly prepared emulsions.

One of the parameters responsible for the quality and precisely associated with the stability of emulsion systems is the size of the dispersed phase droplets. The smaller and more uniform the droplets, the less likely the emulsion will show early destabilization [28]. In the presented study, the mean droplet size (D_(4,3)_) was recorded in the range of 2.2 µm–13.9 µm (Figure 8). Proper immobilization of the droplets guarantees a longer shelf-life of such an emulsion system, primarily by blocking the particles against possible processes as flocculation and, consequently, coalescence. For the tested emulsions, it was found that a greater amount of texture modifier resulted in an increase in the mean droplet size for most cases. The emulsions showing the smallest droplet range (difference between minimum and maximum values) were characterized by the smallest mean droplet size. These emulsions were mainly emulsions with the highest proportion of hemp seed oil in an oil phase, i.e., F13, F14, F15 as well as emulsions F6 and F9. Formulation F3 obtained the widest distribution between the minimum and maximum droplet sizes, with the values 0.357 and 127 µm, respectively. The results of this determination are consistent with the results obtained during the determination of the system stability using the TSI values as well as microscopic evaluation which indicated this emulsion as the one which showed the lowest stability.

## 4. Conclusions

The presented work confirmed that by means of enzymatic modification a new fat can be obtained which can be successfully used as an oil phase of emulsions stabilized by polysaccharides (xanthan gum and scleroglucan). Such a modified fat becomes more attractive because it constitutes target oil phase already equipped with a sufficient amount of emulsifiers. The use of a variable amount of a texture modifier in the proposed emulsion systems did not show clear differences in the stability of the systems. Therefore, in the opinion of the authors, it does not seem justified to use greater amounts of a modifier (above 0.6% *w/w*). Only if the specific rheological properties are required then a higher addition of texture modifier is advisable. Due to the fact that the systems presented in the work are model systems, further development of their composition should be related to a specific purpose, i.e., whether it will ultimately concern a cosmetic, pharmaceutical or food product.

## Figures and Tables

**Table 1:** Formulation of the emulsions.

Phase	Oil Phase		Aqueous Phase		---
Component	MT:HSO Ratio (*w/w*)	Modified Fat Blend (% *w/w*)	Thickener (% *w/w*)	Water (% *w/w*)	Preservative (% *w/w*)
F1	3:1	30.0	0.6	Up to 100.0	1.0
F2	3:1	30.0	0.8	Up to 100.0	1.0
F3	3:1	30.0	1.0	Up to 100.0	1.0
F4	3:2	30.0	0.6	Up to 100.0	1.0
F5	3:2	30.0	0.8	Up to 100.0	1.0
F6	3:2	30.0	1.0	Up to 100.0	1.0
F7	3:3	30.0	0.6	Up to 100.0	1.0
F8	3:3	30.0	0.8	Up to 100.0	1.0
F9	3:3	30.0	1.0	Up to 100.0	1.0
F10	2:3	30.0	0.6	Up to 100.0	1.0
F11	2:3	30.0	0.8	Up to 100.0	1.0
F12	2:3	30.0	1.0	Up to 100.0	1.0
F13	1:3	30.0	0.6	Up to 100.0	1.0
F14	1:3	30.0	0.8	Up to 100.0	1.0
F15	1:3	30.0	1.0	Up to 100.0	1.0

## Data Availability

The data presented in this study are available on request from the corresponding author.
